# Reflection-mode acousto-optic imaging using plane wave ultrasound pulses

**DOI:** 10.1117/1.JBO.26.9.096001

**Published:** 2021-09-01

**Authors:** Lukasz J. Nowak, Wiendelt Steenbergen

**Affiliations:** a University of Twente, Faculty of Science and Technology, Biomedical Photonic Imaging Group, Enschede, The Netherlands

**Keywords:** acousto-optic imaging, plane wave, reflection mode, ultrasound linear array

## Abstract

**Significance:** Performance of an acousto-optic imaging system is limited by light fluence rate and acoustic pressure field distributions characteristics. In optically scattering media, the former determines the achievable contrast, whereas the latter the imaging resolution. The system parameters can be shaped by changing relative positions of ultrasound (US) transducer array and optodes. However, in the case of many potential clinical applications, optimization possibilities in this regard are limited, as a sample is accessible from one side only and using a water tank for coupling is not feasible.

**Aim:** We investigate the possibilities of improving performance of an acousto-optic imaging system operating in reflection mode geometry with linear US array in direct contact with a sample using plane wave instead of focused US pulses.

**Approach:** Differences in acoustic pressure field distributions for various transducer excitation patterns were determined numerically and experimentally. Acousto-optic images of phantoms with and without optically absorbing inclusions were acquired by measuring laser speckle contrast decrease due to the light modulation by plane wave and focused US pulses with different apodization patterns.

**Results:** The residual acoustic pressure field components occupy relatively large volume and contribute to light modulation. Using nonsteered plane wave US pulses instead of focused ones allows one to mitigate their influence. It also allows one to obtain clear two-dimensional reconstructions of light fluence rate maps by shifting transducer apodization along the lateral direction.

**Conclusions:** Using nonsteered plane wave US pulses allows one to achieve better imaging performance than with focused pulses in the assumed system geometry.

## 1 Introduction

In this study, we investigate the possibilities of improvements of imaging performance of a reflection-mode acousto optic imaging (AOI) system with linear ultrasound (US) array using nonsteered plane wave acoustic pulses instead of focused ones. In principle, AOI allows determination of fluence rate distribution of light transmitted through an optically scattering medium by exploiting effects of interaction between the light and an acoustic wave. Due to the induced motion of optical scatterers within the illuminated and insonified volume, the light that is scattered within this region undergoes modulation (the acousto-optic effect).^1^^,^^2^ By determining the ratio of intensities of modulated to nonmodulated light for various acoustic pressure field distributions, the fluence rate map in the medium can be determined. This in turn can be—under certain conditions—used to detect and localize optically absorbing inclusions inside the region of interest.^3^

There have been many approaches to implementing AOI systems described in the literature. Comprehensive reviews of the related studies were presented by Elson et al.,^4^ Resink et al.,^5^ and Gunther and Andersson-Engels.^6^ The presented solutions differ in various aspects, including configuration of optodes, US transducer type and position relative to sample, signal detection means, and combination with other imaging modalities. In this study, we consider a reflection mode AOI, i.e., configuration in which both optodes and US transducer are positioned on the same side of an investigated sample. Such a configuration was previously investigated by Lev et al.,^7^^–^^9^ Hisaka and Saskura,^10^ Kim et al.,^11^ and Hong-Bo et al.^12^ In all of these investigations, the imaging was performed by physically changing the relative positions of the investigated sample and the probe while keeping the US focus constant. Such an approach allows one to directly link the observed changes of the received signal with changes of optical properties within the region of interest.^3^ Here, we impose a condition that the measurement probe should remain stationary relative to the sample during the whole imaging procedure. In other words, the scanning should be performed by electronic US pulse shaping, instead of physically displacing either the probe or the sample. The introduced restrictions are in accordance with practical limitations related to various clinical problems, in which the region of interest is accessibled from one side only, and physical scanning might be not feasible or not desired.

The detected acousto-optic signal is proportional to the product of light fluence rate and acoustic pressure amplitude squared, integrated over the whole volume of interest.^2^^,^^13^ In the considered case of static probe and sample, the fluence rate distribution remains constant, whereas the acoustic pressure field distribution is subject to change to perform scanning and image reconstruction. Typically, focused US pulses are used for light modulation to maximize the achievable acoustic pressure amplitude (increasing signal-to-noise ratio) and minimize the insonified volume (increasing the achievable image resolution). It is then assumed that the acoustic pressure field distribution is entirely concentrated within the desired focal region and by scanning the US focus and measuring AOI signal, the light fluence rate map is reconstructed.^3^ The accurateness of such an assumption, however, strongly depends on transducer type and excitation scheme. In general, focusing of US pulses is achieved either by using specially shaped electroacoustic transducers with constant focus and/or by using a matrix of singly driven transducer elements with excitation delays determined in such a way that constructive interference between all the emitted pulses occurs at the desired focal point. Based on the fundamental principles of operation of any US transducer matrix, one can expect that some residual pressure field components will also be present in the surrounding space—although their amplitudes should be significantly lower than in the focal region.^14^ On the other hand, they may also occupy much greater volume and in this way have significant influence on the determined acousto-optic signal.

In this study, we used a one-dimnsional (1-D) US transducer array for obtaining acousto-optic images. This allowed us to perform line and cross-section AOI scans using electronic pulse shaping and to operate in direct contact with investigated samples. In such a probe, focusing is obtained by applying various delays to the electric pulses driving individual transducer elements. The acoustic pressure field distribution is not limited to focal region only, but the pulses propagate in all directions, forming a complex interference pattern. We hypothesize that this residual acoustic pressure field has significant, negative influence on the obtained AOI signals. This influence could explain, for instance, limited decrease of the speckle contrast difference (SCD) values determined for samples with highly optically absorbing inclusions, as presented in our previous publication.^3^ To test this hypothesis, we developed a new AOI routine, using plane wave instead of focused US pulses.

Using plane wave US pulses for AOI was previously investigated by Laudereau et al.^15^ and Bocoum et al.^16^^,^^17^ The authors also used linear transducer array, but their setups were arranged in transmission-mode geometry. The studies focused on using plane-wave pulses emitted at multiple angles and applying image reconstruction algorithms for improved image quality and data acquisition time. The authors did not consider the influence of residual acoustic pressure field components, despite noting that uncontrolled diffraction effects may limit the system performance when applying additional US wavefront shaping.^16^

In this study, we investigate the influence of the residual acoustic pressure field components (i.e., the non-zero pressure field components outside the desired imaging region) and aim to mitigate their influence on the resulting acousto-optic image in the given reflection-mode configuration with the probe in direct contact with a sample. We determined the expected acoustic radiation characteristics of the used US probe by performing numerical simulations for various excitation patterns and imaging depths. The transducer was also characterized experimentally, by three-dimensional (3-D) field scanning in a water tank, for different emitted US pulses. Next, we performed AOI in the specified configuration, using various phantoms and plane wave and focused US pulses with different apodization patterns. We demonstrate that using nonsteered plane wave US pulses and shifting the transducer apodization pattern it is possible to obtain clear, two-dimensional (2-D) acousto-optic images under all the adopted assumptions. The comparison between the results obtained using plane wave and focused US pulses also indicates that the influence of the residual acoustic pressure field components can be in this way mitigated.

## 2 Materials and Methods

### 2.1 Numerical Simulations of Acoustic Pressure Field Distribution

Numerical simulations of acoustic pressure field distribution generated by a US probe were conducted using k-wave simulation package for Matlab, and in-house Matlab scripts for data postprocessing, analysis, and visualization. We used a 3-D cuboid mesh with dimensions 49 mm (axial) × 49 mm (lateral) × 23.3 mm (elevation), divided into 488×488×232 grid elements. Additional 12 grid elements at each side constituted perfectly matched layer to simulate free-field conditions.

The simulated US linear array consisted of 128 rectangular transducer elements with dimensions 301  μm (lateral; three grid points) × 4 mm (elevation; 40 grid points). The elements were positioned directly next to each other. A constant focus in the elevation direction was set to 25 mm by applying variable excitation delays to the source grid points along the lengths of the elements, using one of the built-in k-wave functions. All the parameters were chosen in such a way as to approximate the geometry and characteristics of an ATL L7-4 US probe, used for the experimental investigations described further. The US pulse frequency was equal to the transducer center frequency, i.e., 5208 MHz. The declared transducer bandwidth is 4 to 7 MHz.

In this study, the acousto-optic signal is determined in terms of SCD, i.e., by calculating the difference of the contrast between two subsequent interference patterns of the detected light—one obtained with and the other without US pulses present—due to the light modulation by acoustic wave. The contrast is defined as a ratio of the standard deviation of pixel values to the mean pixel intensity. The SCD values are proportional to the product of acoustic pressure amplitude squared and fluence rate of light reaching a detector.^13^^,^^18^ Thus, to asses the influence of residual acoustic pressure field components on the detected signal, we introduce a confinity coefficient Φ, defined as

$$\Phi =\frac{{∭}_{V_{i}}{\left|p\right|}^{2}(x,y,z)dx\;dy\;dz}{{∭}_{V_{\mathrm{tot}}}{\left|p\right|}^{2}(x,y,z)dx\;dy\;dz},$$ (1)

where |p|(x,y,z) denotes the acoustic pressure amplitude at the point with spatial coordinates x,y,z, $V_{i}$ is the desired imaging volume, and $V_{\mathrm{tot}}$ is the total insonified and illuminated volume. The confinity coefficient values were determined for focused and nonsteered plane wave US pulses, various imaging depths, and apodization patterns. The desired imaging volume $V_{i}$ is assumed to be a sphere with radius equal to 1 mm and center at the focal point for focused US pulses or a cuboid with volume equal aperture area times 2 mm for plane wave US pulses.

The confinity coefficient Φ can take values between 0 and 1, where 0 corresponds to the situation in which all the pressure field components are outside the desired imaging region, and 1 corresponds to the ideal case with no residual pressure field components present.

### 2.2 Experimental Measurements

The acoustic radiation characteristics of the ATL L7-4 probe were determined experimentally with a fiber-optic hydrophone system (precision acoustics) connected to a data acquisition card (DP105, Agilent Technologies) and a PC computer. The probe was mounted inside a water tank and the scanning was performed using a 3-D translation system controlled with a Matlab script. The mapped volume was a cuboid positioned centrally in front of the transducer, 1 mm away from its facet, with dimensions 49 mm (axial) × 43 mm (lateral) × 15 mm (elevation). The scanning resolution was 1 mm in each direction, and each acquired waveform was averaged over 10 subsequent measurements. The probe was connected to a programmable US scanner (Verasonics Vantage 256), which was triggered from an external function generator (Tektronix AFG3000), providing synchronization with the data acquisition system. Focused and plane wave US pulses were investigated using two different apodization patterns: all 128 elements active and only 64 central elements active. The US frequency was equal to 5208 MHz (central frequency of the probe used), and the transducer elements were excited with single period, bipolar square wave pulses with amplitudes of 85.6 V.

**Fig. 1:**
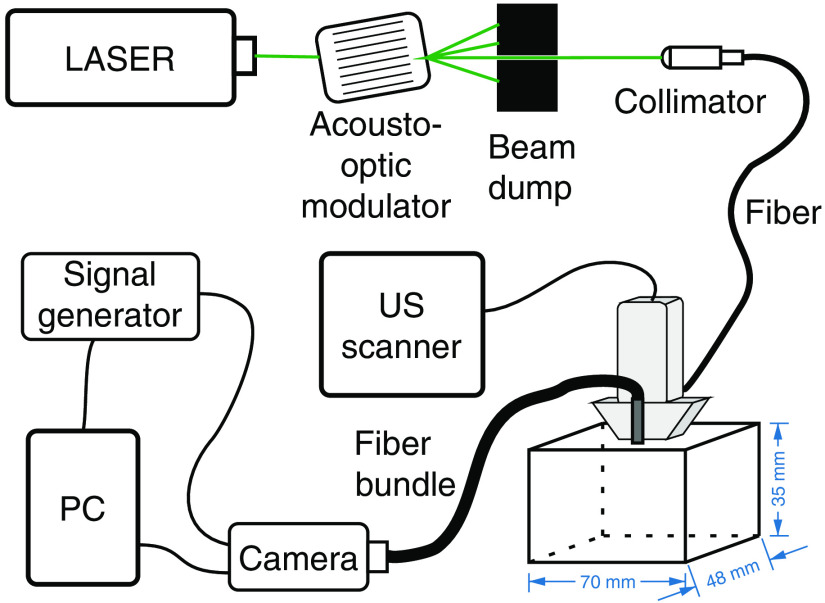
Schematic drawing of the experimental setup used for AOI.

A schematic drawing of the experimental setup used for acousto-optic imaging (AOI) is shown in Fig. 1. It consisted of 532 nm, 6.5 W CW laser (Coherent Verdi 6), and acousto-optic modulator (Gooch & Housego, R23080-3-LTD) for emitting 1  μs light pulses. The light was transmitted to the investigated samples using a 200-μm multimode fiber, and then detected using a circular fiber bundle with a diameter of 4 mm, projecting the optical intereference patterns on a sensor of a CCD camera (Allied Vision Stingray F-125B).

The transmitting and receiving fibers facets were positioned 35 mm away from each other, on both sides of an US linear array (elevation direction, centrally). The US transducer was 128 element ATL L7-4 probe. We used a detection scheme based on SCD measurements described in our previous studies.^3^^,^^19^ We captured pairs of subsequent camera frames, first exposed to a series of laser pulses, and the subsequent one, for which identical number of laser pulses were accompanied by focused or plane wave US pulses. The US and laser emissions were synchronized in such a way that the illumination occurred when the acoustic wave reached its focus or the desired imaging depth. Two different types of AOI scans were performed. First, 1-D axial scans along the central axis of the probe were conducted for both focused and plane wave US AOI, to compare the results obtained using both imaging modalities. Next, we investigated the possibilities of obtaining 2-D AOI scans using plane wave US pulses with different apodization patterns. In all the cases, the calculated SCD values were averaged over 20 subsequent frame pairs captured.

The measurements were conducted using cuboid phantoms casted from polyvinyl chloride plastisol (PVCP) with addition of 3.5 mg/ml of titanium dioxide, with dimensions of $70\times 48\times 35\;{\mathrm{mm}}^{3}$. Two phantoms were used: one homogeneous and one with a cylindrical, optically absorbing inclusion (casted from the same material with addition of black ink) with diameter 3 mm, located centrally, 5 mm below the surface, oriented along one of the edges. The optical and acoustic properties of the phantoms were designed to be within tissue-like value range, with the optical scattering coefficient $\mu_{s}=45\;{\mathrm{cm}}^{-1}$, optical absorption coefficient $\mu_{a}$ negligible for PVCP without ink, and equal to approximately $17\;{\mathrm{cm}}^{-1}$ for inclusion, and speed of sound equal 1700 m/s.

## 3 Results

**Fig. 2:**
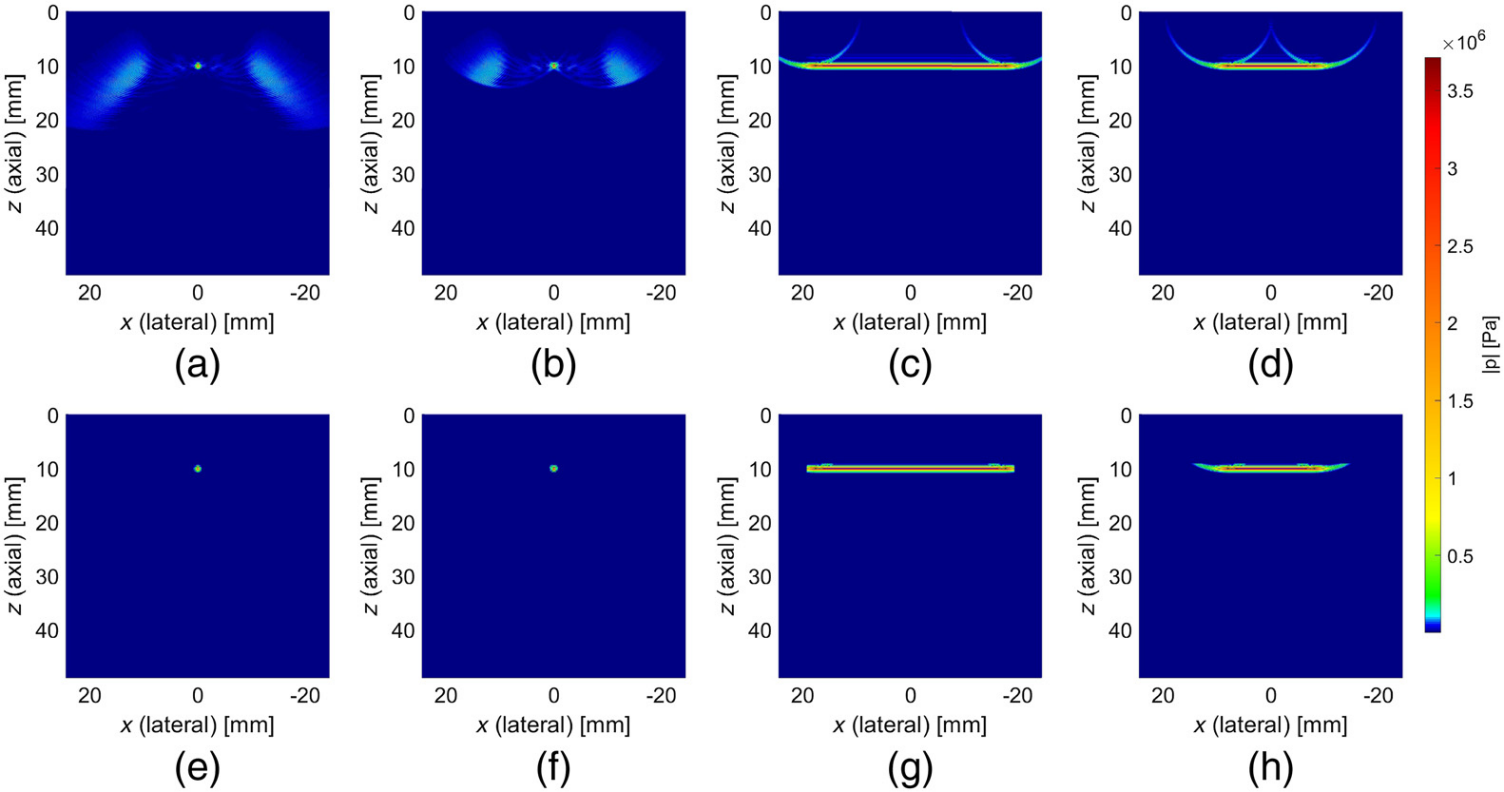
Results of the numerical simulations of acoustic pressure field distributions in axial-lateral plane for various US pulse types: (a) focused at 10 mm, all 128 elements active; (b) focused at 10 mm, 64 central elements active; (c) plane wave at 10 mm, all 128 elements active; and (d) plane wave at 10 mm, 64 central elements active. (e)–(h) The cross-sections of pressure maps obtained by masking the corresponding distributions to simulate the idealized cases, without any residual pressure field components.

Examples of results of numerical simulations of acoustic pressure field distributions obtained for focused and plane wave US pulses with different apodization patterns are shown in Figs. 2(a)–2(d). Figures 2(e)–2(h) show cross-sections of the same distributions obtained by masking only the desired imaging regions (i.e., a sphere with radius equal to 1 mm and center at the focal point for focused US pulses or a cuboid with volume equal aperture area times 2 mm for plane wave US pulses). Differences between the corresponding plot pairs [i.e., Figs. 2(a)–2(e), Figs. 2(b)–2(f), Figs. 2(c)–2(g), and Figs. 2(d)–2(h)] constitute the residual pressure field components.

**Fig. 3:**
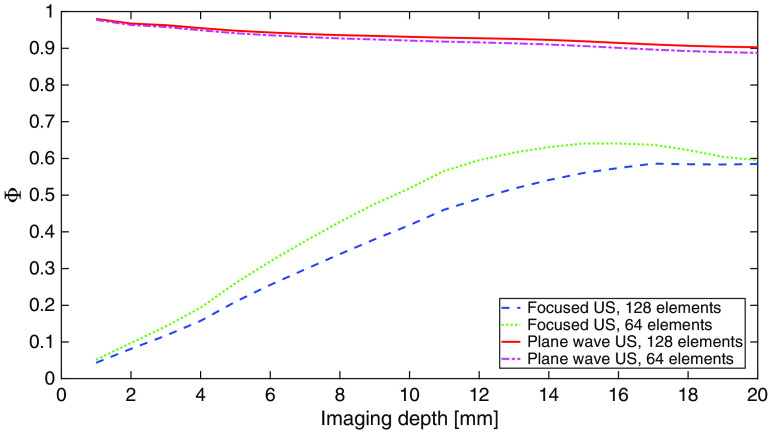
Confinity coefficient values determined as functions of imaging depth based on the results of numerical simulations conducted for different US pulse types.

The numerical simulations were conducted for focused and plane wave US pulses, with all 128 transducer elements or only 64 central elements active, and for imaging depths between 1 and 20 mm, with 1 mm resolution. The masked counterpart representing the desired imaging volume was also determined in every single case, and in this way, the confinity coefficient values were determined as functions of the imaging depth, accordingly to the introduced Eq. (1). The results are shown in Fig. 3. The confinity coefficient is significantly higher for plane wave than for focused US pulses, with relatively low differences related to the apodization numbers.

**Fig. 4:**
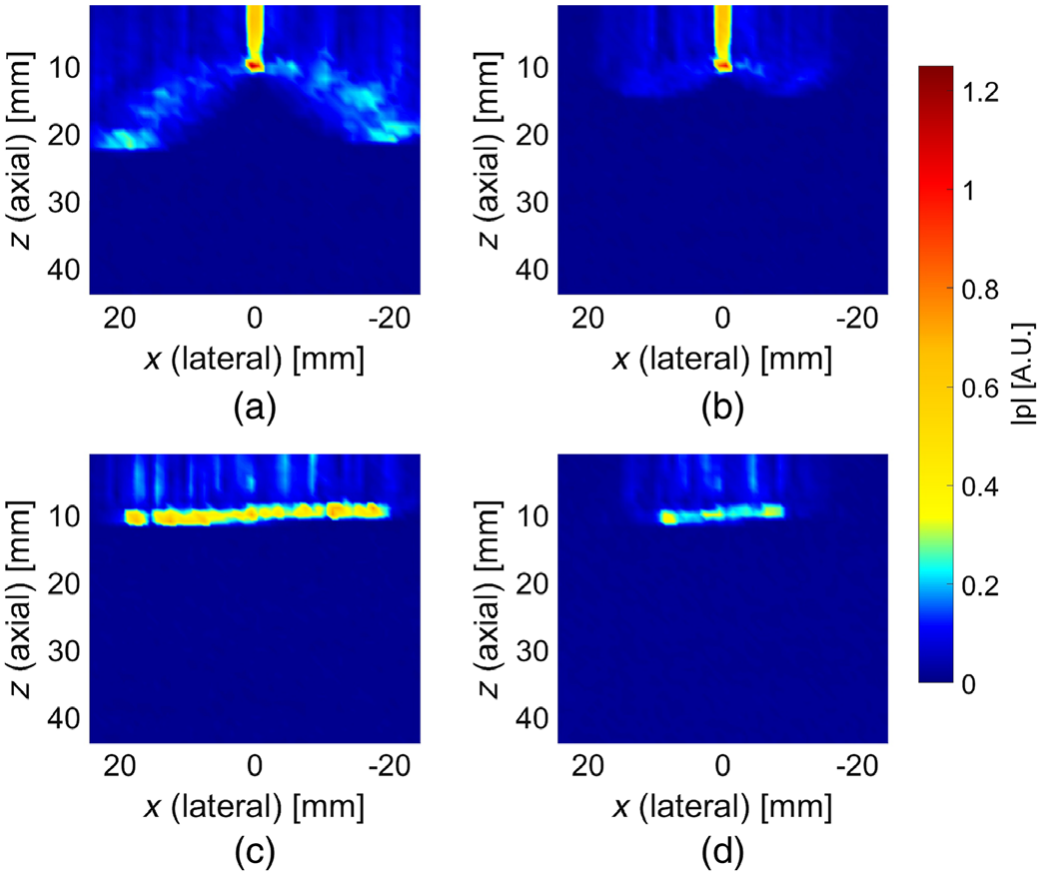
Acoustic pressure field distributions determined experimentally for various US pulse types: (a) focused at 10 mm, all 128 elements active; (b) focused at 10 mm, 64 central elements active; (c) plane wave at 10 mm, all 128 elements active; and (d) plane wave at 10 mm, 64 central elements active.

Cross-sections of acoustic pressure field distributions determined experimentally for focused and plane wave US pulses are shown in Fig. 4. Similarly as in the results of numerical simulations shown in Fig. 2, the assumed imaging depth was equal to 10 mm, and two different apodization patterns, with all 128 transducer elements and only 64 central elements active, were considered. The data present maximum absolute pressure values measured within the 1  μs time window centered at the calculated time of focus and averaged over 10 subsequent pulses, for every single hydrophone position. The experimental data expose more residual pressure field components than the numerically determined distributions. This is also reflected in the confinity coefficient values determined for the experimental data and the imaging depth 10 mm, presented in Table 1, which are significantly lower than the corresponding values calculated numerically.

**Table 1:** Confinity coefficient values determined experimentally at imaging depth of 10 mm.

	128 elements active	64 central elements active
Focused US	0.081	0.08
Plane wave US	0.643	0.354

Based on the results of numerical simulations shown in Fig. 2, the predicted ratio of maximum acoustic pressure amplitude of US pulse focused at 10 mm to analogous value calculated for plane wave excitation and identical depth was equal to 4.54 when all 128 transducer elements were used. For 64 central transducer elements active, this ratio was 4.22. Analogous values obtained from the experimental data shown in Fig. 4 were equal to 1.58 and 2.69 for 128 and 64 transducer elements active, respectively.

**Fig. 5:**
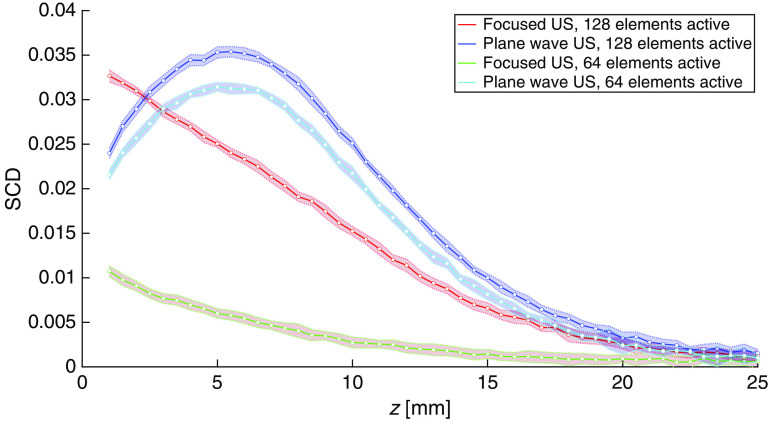
SCD values determined as functions of imaging depth along the central axis of the probe for the homogeneous phantom, using focused and plane wave US pulses with all 128 transducer elements or only 64 central elements active.

Figure 5 shows the SCD values as functions of imaging depth determined experimentally using focused and plane wave US pulses with two different apodization patterns, for the phantom without any optically absorbing inclusions. The shapes of the obtained curves are significantly different, although the mapped light fluence rate distribution was identical for all the measurements. Those differences must therefore result from variations in acoustic pressure field distributions used for light modulation.

**Fig. 6:**
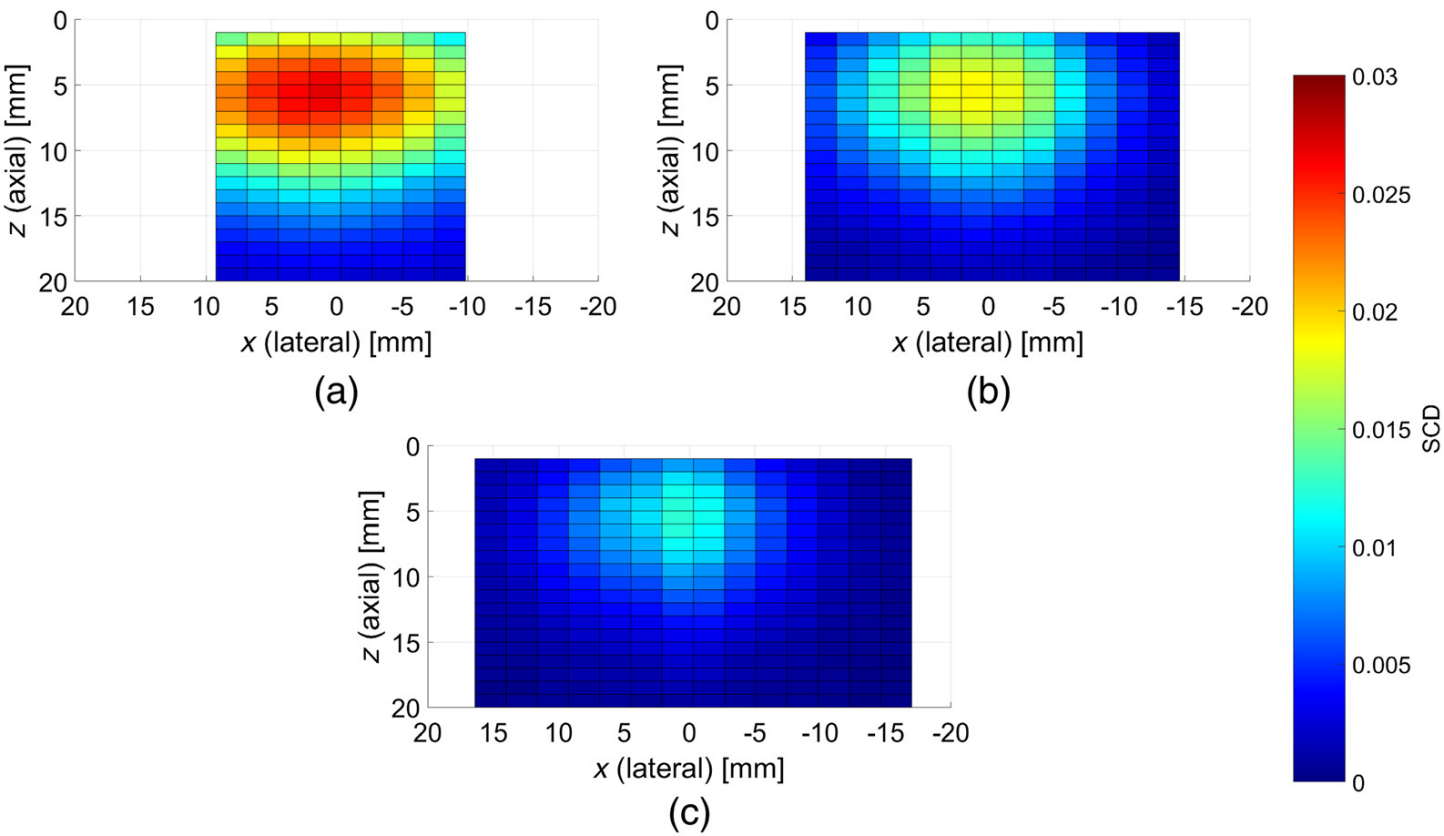
SCD values determined in axial-lateral plane inside the phantom without any inclusions. The images were obtained using plane wave US pulses and apodization numbers of: (a) 64, (b) 32, and (c) 16 active elements. Scanning in the lateral direction was performed by shifting the apodization along the transducer array with eight elements step size.

We performed 2-D AOI using plane wave US pulses by shifting the apodization pattern of simultaneously excited transducer elements along the 1-D US array. The results obtained for the phantom without any optically absorbing inclusion, three different apodization numbers (with 64, 32, and 16 elements active), and eight element step size are shown in Fig. 6. In all the cases, a clear local maximum is visible at the center, ∼5  mm below the surface. The determined SCD values decrease together with decreasing number of excited transducer elements. Also, higher apodization numbers correspond to fewer possible steps along the lateral direction and thus limited field of view.

**Fig. 7:**
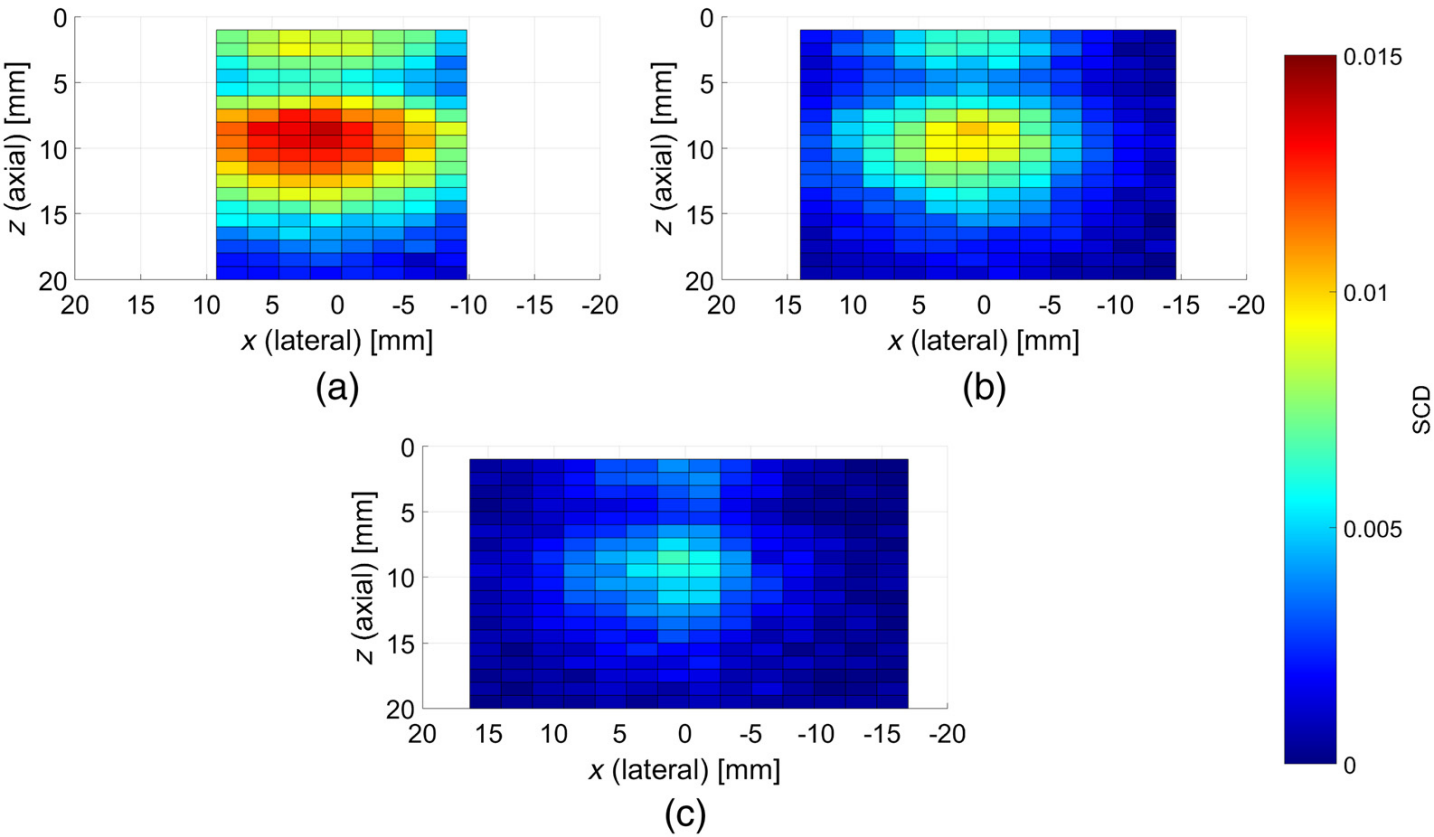
SCD values determined in axial-lateral plane inside the phantom with a cylindrical, optically absorbing inclusion with diameter of 3 mm, located 5 mm below the surface and oriented along the lateral direction, centrally below the probe. The images were obtained using plane wave US pulses and apodization numbers of: (a) 64, (b) 32, and (c) 16 active elements. Scanning in the lateral direction was performed by shifting the apodization along the transducer array with eight elements step size.

Figure 7 shows analogous results of 2-D plane wave AOI scans obtained for the phantom with the cylindrical, optically absorbing inclusion with diameter 3 mm, located centrally 5 mm below the surface and oriented along the lateral direction (in the x-z plane). The determined SCD distributions reveal in this case a clear local minimum at the depth corresponding to the position of the inclusion. The obtained SCD values are significantly lower compared with the case of phantom without any inclusions, as shown in Fig. 6.

## 4 Discussion

Results of the numerical simulations of acoustic pressure field distributions obtained for focused and plane wave US pulses and different apodization patterns reveal in each case presence of residual components, occupying the volume outside the desired imaging regions. Although no analytical solutions exist for the general case of near-field acoustic radiation characteristics of US transducers, the obtained distributions are consistent with the intuition based on the principle of operation of such devices. In a 1-D linear array, the focusing is achieved by applying varying delays to the electric pulses exciting individual transducer elements in such a manner that a constructive interference is achieved at the desired focal point. Thus, if the focal point is located along the central axis of the probe, the lateral-most transducers will be excited as first, and the central elements as the last ones in order to compensate for the distance differences. In the considered case, all the transducer elements are identical and have the same radiation characteristics. For that reason, the distinct (in terms of the achieved pressure amplitude) focal points is surrounded by characteristic lobes, extending furthermost at the extreme locations along the lateral direction. This phenomenon is mitigated—but still clearly visible—in the case of reduced apodization number.

In the case of plane wave US pulses, all active transducer elements are excited at the very same moment. The assumed imaging volume (i.e., the volume in which all the light modulation is expected to occur) in such a case is a cuboid with cross-section equals the US array apodization area and height (axial direction) equals to the US pulse length. An important conclusion based on the described principle of operation only is that the residual pressure field components cannot precede the propagating region of interest. The results of the numerical simulations shown in Fig. 2 predict that the components outside the desired imaging volume take a shape of two semicircles adjacent to the main wavefront edges and following it, occupying relatively smaller volume than in the case of the focused US pulses.

The acoustic pressure field distributions obtained experimentally, as shown in Fig. 4, are similar to the results of numerical investigations presented in Fig. 2 in the parts corresponding to the leading field components. This confirms the general predictions regarding electronic pulse shaping. However, there are also significant discrepancies in the form of extended trailing field components. The nature of the discrepancies suggests that they might result from unpredicted extension of the emitted US pulses. This could be caused by nonideal impulse responses (limited bandwidths) of the transducer elements and/or cross-talk between them, enforced by high voltage amplitudes and apodization numbers used. Neither of these phenomena was included in the simulations, and both of them would result in degradation of the pressure field concentration. This could also explain the elongated shape of the focus in surface shown in Figs. 4(a) and 4(b), as due to the symmetry of the excitation delays a constructive interference would occur at the central axis. Such hot spots do not appear in the case of plane wave imaging, as all the transducer elements are excited at the very same moment.

To quantify and investigate the possible influence of residual acoustic pressure field components on the acousto-optic signal, we have introduced the confinity coefficient Φ, defined with Eq. (1). It describes the ratio of the acoustic pressure field components confined within the desired imaging region to the pressure field distribution within the whole insonified volume. It is defined for specific US transducer excitation pattern and imaging coordinates. In general, the higher value of confinity coefficient is, the better AOI resolution can be achieved. Numerical simulations predicted that for nonsteered plane wave US the Φ value could be close to 1, slowly decaying with increasing imaging depth. For focused US pulses, this quantity is much lower and reveal reverse relationship with the imaging depth. Also, a local maximum or saturation effect is visible for focused US in Fig. 3. This is due to the fact that for the deepest considered locations the residual pressure field components begin to fall outside the assumed medium boundaries. The Φ values determined from experimental measurements are significantly lower, compared with the results of simulations, which is directly related to the observed presence of excessive residual pressure field components following the main wavefront. Also in this case, the values calculated for plane wave pulses are several times greater than the ones obtained for focused US. However, one should notice that the confinity coefficientwise advantage of plane wave US pulses over focused pulses is achieved at the cost of much greater imaging volume in the former case.

We compared the imaging performance achieved using focused and plane wave US by performing 1-D AOI scans inside an optically and acoustically homogeneous phantom, as shown in Fig. 5. In such a medium, for the assumed system configuration, the fluence distribution of light transmitted between the optodes takes a banana-like shape.^20^ Thus, the SCD values determined along the probe’s center axis should reveal a global maximum followed by a steady decay. Indeed, such results are observed for plane wave US pulses. Also, the determined values in this case are relatively slightly dependent on the apodization number used. The SCD values determined using focused US pulses uniformly decrease with depth and reveal strong dependence on the apodization pattern—the results obtained using 64. active transducer elements are approximately three times lower than the ones obtained for all 128 elements active. Such discrepancies in signal levels could not be explained by the differences in acoustic pressure amplitudes at the focal point since—as it is clearly visible in both simulation and experimental results shown in Figs. 2 and 4—they do not vary significantly. Also, the expected focal volume in the case of focused US pulses should not change significantly with the aperture size used. Such observations indicate that the residual pressure field components play an important role in light modulation when using focused US pulses in the described system configuration. In the case of plane wave US pulses, all active transducer elements are excited at the very same moment, and thus the residual field components are following—but never preceding—the main wavefront and are concentrated within a smaller volume, compared with focused pulses emitted over a longer time period. Under such conditions, as shown in Fig. 5, it is possible to visualize the expected maximum of the fluence rate distribution of the detected light.

Mitigating the influence of residual pressure field components using plane wave US pulses is achieved by significantly extending the region of interest in which the light modulation is expected to occur. In the assumed system configuration, the extension takes place along the lateral axis, and thus one might expect that the imaging resolution along this direction will be impaired. We investigated the possibilities of 2-D AOI using nonsteered plane wave US pulses. The scanning in the lateral direction was performed by shifting the apodization pattern along the US transducer array. The results shown in Fig. 6 are consistent with the expected general shapes of cross-sections of banana-shaped fluence rate distributions between the optodes positioned centrally and perpendicularly to the US array. There is a clear dependence between the achievable SCD values and the apodization numbers used. It was not the case for the results shown in Fig. 5; however, here the apodization numbers are much lower, to enable cross-section scanning. The more transducer elements were active, and the higher contrast was observed. On the other hand, increasing the apodization number also decreases the field of view, as less steps along the lateral direction can be performed. There are no significant differences in the shapes of the obtained distributions shown in Figs. 6(a)–6(c).

When homogeneous phantom was replaced with the phantom containing an optically absorbing inclusion, the SCD distributions determined using 2-D plane wave US AOI scans revealed a distinct local minimum corresponding to the location of the inclusion, as shown in Fig. 7. Such feature in the reconstructed light fluence rate maps is a direct indication of presence and location of an optically distinct region. Also in this case, the shapes of the obtained distributions do not reveal any significant differences due to various apodization numbers used, and the achievable SCD values were higher when more transducer elements were simultaneously active.

All the presented SCD distributions were obtained by averaging 20 subsequent measurements for given imaging coordinates. The camera frame rate was set to 10 frames per second. This translates to 2 s imaging time required for every data point, which gives ∼100  s for line scans shown in Fig. 5, and between 5 min and 20 s and 9 min and 20 s for cross-section scans shown in Figs. 6 and 7. However, as the camera exposure time was set to 50 ms, the frame rate could in principle be increased, yielding the decrease of the required imaging time by more than 10 times. Here, the data acquisition rate was chosen arbitrarily to prevent potential transmission errors, as during static laboratory experiments the imaging time was not crucial. Still, the presented technique has many improvement possibilities in this regard, which are related to purely technical issues.

## 5 Summary

We investigated the possibilities of using nonsteered plane wave US pulses for 1-D and 2-D AOI in reflection mode geometry. The goal was to improve the imaging performance in the assumed system configuration by mitigating the influence of residual acoustic pressure field components. We compared pressure field distribution patterns determined with numerical simulations and experimental measurements for plane wave and focused US pulses with different apodization patterns. We also introduced the confinity coefficient, describing the extent to which the pressure field is confined within the desired imaging volume, and calculated its value as functions of depth for the considered US pulses. The results revealed advantage of plane wave pulses, which is achieved at the cost of significantly extending region of interest in the lateral direction. The influence of residual pressure field components was demonstrated by comparing the SCD values determined experimentally as functions of depth inside a homogeneous, optically scattering medium using plane wave and focused US pulses. We also showed that it is possible to perform 2-D AOI scans and to obtain clear cross-sectional light fluence rate maps using plane wave US pulses and shifting apodization along the transducer array. The resolution in the axial dimension is determined by the US pulse length and in the considered case allowed to obtain clear local minimum corresponding to the location of a 3-mm diameter optically absorbing inclusion. The lateral resolution depends on the US transducer apodization pattern. Higher apodization numbers limit the available field of view but improve image contrast. In case when the fluence rate of the detected light reveals simple structure with single local maxima along the lateral cross-sections, the shapes of the reconstructed distributions do not depend on the numbers of active US transducer elements used, and the imaging resolution is determined by the step size.

## Code, Data, and Materials Availability

The datasets generated within the framework of the described study are available from the corresponding author upon request.
